# Insights into oral microbiome and colorectal cancer – on the way of searching new perspectives

**DOI:** 10.3389/fcimb.2023.1159822

**Published:** 2023-04-12

**Authors:** Anna Kudra, Damian Muszyński, Bartosz Kamil Sobocki, Alessandro Atzeni, Ludovico Carbone, Karolina Kaźmierczak-Siedlecka, Karol Połom, Leszek Kalinowski

**Affiliations:** ^1^ Scientific Circle of Studies Regarding Personalized Medicine Associated with Department of Medical Laboratory Diagnostics, Medical University of Gdansk, Gdansk, Poland; ^2^ Scientific Circle of Oncology and Radiotherapy, Medical University of Gdansk, Gdansk, Poland; ^3^ Institut d’Investigació Sanitària Pere Virgili (IISPV), Reus, Spain; ^4^ Universitat Rovira i Virgili, Departament de Bioquímica i Biotecnologia, Unitat de Nutrició, Reus, Spain; ^5^ Centro de Investigación Biomédica en Red Fisiopatología de la Obesidad y la Nutrición (CIBEROBN), Institute of Health Carlos III, Madrid, Spain; ^6^ Department of Medicine Surgery and Neuroscience, University of Siena, Siena, Italy; ^7^ Department of Medical Laboratory Diagnostics – Fahrenheit Biobank BBMRI.pl, Medical University of Gdansk, Gdansk, Poland; ^8^ Department of Surgical Oncology, Medical University of Gdansk, Gdansk, Poland; ^9^ BioTechMed Centre, Department of Mechanics of Materials and Structures, University of Technology, Gdansk, Poland

**Keywords:** colorectal cancer, oral microbiome, bacterial virulence factors, biofilm, periodontitis

## Abstract

Microbiome is a keystone polymicrobial community that coexist with human body in a beneficial relationship. These microorganisms enable the human body to maintain homeostasis and take part in mechanisms of defense against infection and in the absorption of nutrients. Even though microbiome is involved in physiologic processes that are beneficial to host health, it may also cause serious detrimental issues. Additionally, it has been proven that bacteria can migrate to other human body compartments and colonize them even although significant structural differences with the area of origin exist. Such migrations have been clearly observed when the causes of genesis and progression of colorectal cancer (CRC) have been investigated. It has been demonstrated that the oral microbiome is capable of penetrating into the large intestine and cause impairments leading to dysbiosis and stimulation of cancerogenic processes. The main actors of such events seem to be oral pathogenic bacteria belonging to the red and orange complex (regarding classification of bacteria in the context of periodontal diseases), such as *Porphyromonas gingivalis* and *Fusobacterium nucleatum* respectively, which are characterized by significant amount of cancerogenic virulence factors. Further examination of oral microbiome and its impact on CRC may be crucial on early detection of this disease and would allow its use as a precise non-invasive biomarker.

## Graphical Abstract

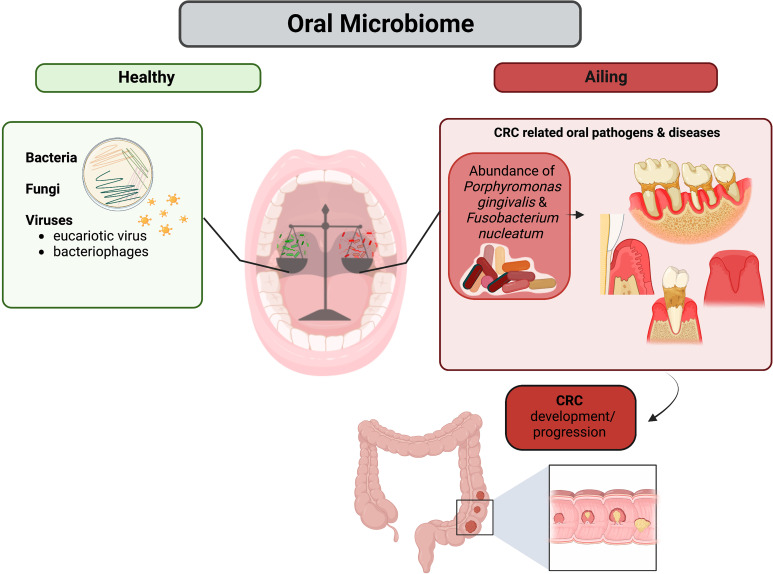

## 1 Introduction

Nowadays, colorectal cancer (CRC) still represents one of the most commonly diagnosed types of cancer worldwide. According to the GLOBOCAN – Global Cancer Statistics 2020, colorectal cancer was assessed as the third (10%) most commonly diagnosed cancer globally and the second cause of death (9.4%) (Sung et al., 2021). Moreover, research work “Global colorectal cancer burden in 2020 and projections to 2040” reported that the number of new CRC cases will increase by 2040 approximately up to 3.2 million cases, causing a huge impact on global healthcare system (Yue and Pengfei, 2021). The highest rates are in the most developed countries, suggesting that diet (i.e., low intake of dietary fiber and diet with high content of saturated fatty acids, consumption of red meat), sedentary lifestyle and obesity, as well as environmental risk factors play a major role in cancer development (Geicho et al., 2015; Mármol et al., 2017; Kwong et al., 2018; Thanikachalam and Khan, 2019; Kaźmierczak-Siedlecka et al., 2020a; Sninsky et al., 2022). Moreover, alterations in “healthy” microbiome and microbial-derived metabolites (known as part of metabolome) might, among others, simulate local inflammatory response and increase the risk for CRC. Interestingly, in 2021 Wang et al. described age at diagnosis, male gender, poor oral hygiene, as well as altered salivary abundance of *Desulfovibrio desulfuricans* (anaerobic bacteria) as predict risk factors of CRC and incorporated them in a clinical nomogram (Wang Y et al., 2021). Over the past decade, dysbiotic changes in oral microbiome allowed better understanding of the pathogenesis of oral cancers and others distal organs disorders. For instance, the *Porphyromonas gingivalis* (Gram-negative oral anaerobe) is, on the one hand, a major periodontopathic pathogen involved locally in development of oral squamous cell carcinoma; on the other hand, it participates also in other localization, for instance in pancreatic carcinogenesis (Mysak et al., 2014; Öğrendik, 2017; Olsen and Yilmaz, 2019; Zhou and Luo, 2019). Another example is *Candida albicans*, whose infection induces several cancerous hallmarks, such as activation of proto-oncogenes, induction of DNA damage and overexpression of inflammatory signaling pathways, contributing to both oral cancer progression and gastric carcinogenesis (Engku Nasrullah Satiman et al., 2020; Zhong et al., 2021).

Considering this and other evidence, oral microbiome may distally affect the development/progression of CRC. Therefore, in this review the link between oral microbiome and CRC occurrence has been discussed. We presented the virulence factors of oral pathogens associated with CRC and the significance of creating biofilm by microorganisms. Moreover, we described the usage of oral microbiome as biomarker to detect CRC.

## 2 Oral microbiome in healthy individuals

Oral cavity represents the second mostly inhibited by microbes area of the human body (Verma et al., 2018). Approximately 772 prokaryotic species are comprised in the extended Human Oral Microbiome Database (eHOMD) (Verma et al., 2018). Six extensive phyla have been distinguished using 16S rDNA profiling, Firmicutes, Actinobacteria, Proteobacteria, Fusobacteria, Bacteroidetes and Spirochaetes (Verma et al., 2018). Different species of bacteria populate main seven regions of human mouth, i.e., gingival sulcus, cheek, attached gingiva, teeth, tongue, lip, hard palate, and soft palate (Dewhirst et al., 2010). Microbiome inhabiting those areas is not hazardous for our health as long as it does not colonies other areas of the human body. Although these bacteria are present in the oral cavity as part of its microbiome, more recent attention has focused on the correlation with several diseases, such as periodontitis, gingivitis, and dental caries, which have a definite connection to modifications in the oral microbiota (Willis and Gabaldón, 2020). These studies were carried out on so called “WEIRD” populations. The acronym referred to Western, Educated, Industrialized, wealthy, and Democratic countries, indicating a bias in psychology research toward these cultures, which at the time made up around 13% of the world’s population (Willis and Gabaldón, 2020). Nevertheless, even within “WEIRD” populations oral microbiome may differ since there are numerous factors which could affect environmental conditions in oral cavity, such as overall hygiene, food and water intake, individual composition of saliva, lifestyle, and many others (Zaura et al., 2014).

The first study assessing the composition of oral mycobiota (fungal part of oral microbiota) in healthy individuals has been published in 2010 (Ghannoum et al., 2010). The authors reported that the most frequently identified genera of fungi were *Candida*, *Cladosporium*, *Aurebasidium*, *Saccharomycetales*, and *Aspergillus*. Moreover, *Fusarium* and *Cryptococcus* were also identified (Ghannoum et al., 2010). Interestingly, interactions between the bacterial and fungal fraction of the oral microbiome have been described. For instance, *C. albicans* initiates alterations of virulence factors of *Streptococcus mutans*, which is known as major cariogenic pathogen; notably, exoenzyme GtfB of *S. mutans* causes the formation of exopolysaccharide matrix and *C. albicans* intensifies that effect consequently contributing to growth of dental biofilm (Baker et al., 2017). The interactions between *C. albicans* and *F. nucleatum* as well as *C. albicans* and *Streptococcus oralis* were also observed (Baker et al., 2017). The effects of interactions between microbes contribute to the development of local and distal diseases. As it was mentioned above, *Candida* genus normally resides in an oral cavity with no observed pathological changes. However, in case of for instance: infections, immune-related disorders, diabetes, antibiotics taking, low production and secretion/flow of salivary, poor oral hygiene, and wearing dentures it caused oral candidiasis (Hu et al., 2019; Rodrigues et al., 2019; Martorano-Fernandes et al., 2020). Therefore, oral mycobiota seems to be significant in the context of CRC patients who are often treated with radiotherapy, chemotherapy and are at higher risk of *Candida*-associated diseases development. It should also be noted that dysbiotic alterations of intestinal fungal community is found in CRC patients. It is observed by, among others, reduced amount of *Saccharomyces cerevisiae* (Coker et al., 2019). Nevertheless, the data regarding the link between oral mycobiota and CRC is still strongly limited and that is associated with several reasons, for instance more complicated methodology than in case of bacteria analysis (Kaźmierczak-Siedlecka et al., 2020b).

Another important component of the oral cavity human microbiome is the oral virome, which, in healthy individuals, includes eukaryotic viruses as well as bacteriophages. Particularly, eukaryotic viruses are Herpesviriade, Papillomaviridae, and Anelloviridae, whereas the most common bacteriophages are Siphoviridae, Myoviridae, and Podoviridae. Oral virome is stable for a long period and, similarly as in case of bacterial oral microbiome, oral virome is different between individuals (Baker et al., 2017).

## 3 The impact of diet and other lifestyle-related factors on oral microbiome

Several studies focused on the impact of diet on the structure of human oral microbiome have recently emerged. A main risk factor, contributing to the growth of pathogenic bacteria as well as development of caries and biofilm expansion, seems to be an excessive intake of carbohydrates, especially sucrose (Sheiham, 2001; Moynihan and Petersen, 2004). Most common pathogenic bacteria use carbohydrates in processes of fermentation turning them into acidic products, which ultimately lead to the development of caries. Several lines of evidence suggest that richness of variety of oral bacteria can differ depending on sugar intake (Bostanci et al., 2021). In 2014 researchers attempted to evaluate whether long-term modifications in dietary habits have a significant impact on microbiota in saliva. Authors examined microbiome as well as metabolic profiles of 161 healthy patients who declared to follow a vegan, omnivore or ovo-lacto-vegetarian diet. After analysis of sequenced amplicons from the 16S rRNA gene’s V1-V3 regions and the analysis of salivary metabolome through 1H-NMR and GC-MS/SPME, researchers came to the conclusion that long-term eating habits have negligible influence on salivary microbiome composition (De Filippis et al., 2014). Another interesting study results were presented by Zaura et al. where in order to better understand the ecobiological variety of the salivary ecosystem and the relationships between the salivary microbiome, salivary metabolome, and host-related biochemical salivary parameters, 268 healthy patients were evaluated after overnight fasting. This study revealed a correlation between daily protein intake and salivary pH, which indicates that diet may affect the environment in the oral cavity *via* altering salivary pH (Zaura et al., 2017).

Translational research projects, such as that conducted by Nagihan Bostanci in the 2021, have investigated smoking habits on oral microbiome composition. Patients were asked to answer a questionary describing them as “daily smoker”, “occasional smoker”, “former smoker”, and “never smoker”. Saliva samples were collected using SalivaGene Collector which enabled the quantification of DNA concentration using the Quant-iT Picogreen dsDNA test. Results demonstrated that smoking does not contribute significantly to the heterogeneity of oral microbiome, while a slight elevation of richness of oral bacteria species was observed in daily smokers in comparison with never smokers (Bostanci et al., 2021). According to research conducted by Le et al. consuming considerate amounts of alcohol may also influence oral microbiota. In this study researchers were looking for correlation between alcohol abuse and the diurnal variation of salivary microbiome. 53 patients were tested for alterations in composition of oral microbiota and individual taxon abundance by 16S rRNA gene sequencing. Results stated that alcohol usage increased the richness of the salivary microbiome while decreasing its evenness. The oral microbiota composition altered dramatically in patients with alcohol abuse history. Furthermore, specific taxa, such as *Actinomyces, Leptotrichia, Sphaerochaeta*, and *Cyanobacteria*, which are known for their negative impact on homeostasis in oral cavity, were enriched in the research group (Li X. et al., 2022). The following study focused on the potential impact of sleep on the oral microbiota. A 16S rRNA gene sequencing analysis was performed to determine alterations. Material for this study was obtained with an Isohelix swab from several locations in the mouth cavity before and after sleep. As comparison to the pre-sleep schedule, the Chao1 index for samples from the buccal mucosa and gingival mucosa, as well as the Shannon index for buccal mucosa samples, were both significantly higher. Variations between before and after sleep may be due to changes in numerous variables influencing oral microbiome during sleep. For instance, during sleep, IgA concentrations rise, saliva pH lowers, and the temperature inside the mouth cavity decreases (Sotozono et al., 2021).

## 4 The alterations of oral microbiota in CRC

### 4.1 Dental biofilm and its significance

Human digestive system homeostasis is possible thanks to the symbiotic relationship between the bacteria inhabiting the gastrointestinal tract and the host. Despite being in different anatomical locations, the colon and the mouth cavity are both heavily populated by different microbiota (Koliarakis et al., 2019). Occasionally, symbiotic relationship occurring between bacteria and hosts oral cavity can be disrupted due to various factors such as neglect of hygiene, decreased production of saliva, change in diet, or weakened immune system. Such events may lead to extensive build-up of dental biofilm inside oral cavity on different surfaces, but mostly on uneven areas of teeth like pits and fissures, in interdental gaps and near gingiva (Chenicheri et al., 2017). Dental biofilm causes an increase of microbes’ drugs resistance and host immune system functioning impairment. A complex multi-microbial community known as a dental biofilm is encased in a polymeric matrix that helps community expansion, bypasses the host’s defenses and encourages colonization of mucosal surfaces through adhesion mediated by different glycoproteins (Chenicheri et al., 2017). Dental biofilms in diseases inside the oral cavity mostly develop in three stages (Kolenbrander et al., 2010). First stage begins right after cleaning when the surface of teeth is exposed to salivary component consisting of alpha-amylase, proline-rich proteins, mucins, and other proteins, with a glycoprotein covering layer leading to create pellicle. Between various glycoproteins, salivary components, and the tooth surface, a variety of interactions such as hydrogen bonds, acid-base interactions, calcium bridges, hydrophobic interactions van der Waals, and electrostatic interactions take place, causing conformational changes in the proteins forming the pellicle. Through strong interactions, bacteria may stay connected not only to the target surface but also to each other, promoting primary colonizers such as *Lactobacillus acidophilus*, *Veillonella*, *Neisseria*, *Actinomyces* spp. and *Streptococcus* spp. to occupy new areas. Mostly Gram-positive species colonize the supragingival surface of teeth while Gram-negative species colonize subgingival surface. As a byproduct of their carbohydrate fermentation process, oral streptococci create lactic acid, resulting in a fast reduction of environmental pH. *C. albicans* grows as yeast at acidic pH levels and as hyphal at alkaline pH levels, hence extracellular pH can affect hyphal development and consequently dental biofilm formation. For the dental biofilm to grow, communication among the interspecies in the plaque is required. Later, due intensive usage of substrates by primary colonizers, bacteria of the genus *Veillonella* can no longer obtain glucose, and they begin to break down lactic acid into acetic and propionic acid, gaining energy for growth and development.

### 4.2 Virulence factors of oral pathogens related to CRC development/progression

Herb Brody defined colorectal cancer as “a disease of modernity”, highlighting the existing relationship between several environmental determinants and the incidence of colorectal cancer (Brody, 2015). Most pathogenic bacteria colonizing oral cavity may contribute to development or progression of CRC *via* its cancerogenic metabolites as well as other virulence factors. Many studies tried to examinate how different species of bacteria participate in carcinogenic processes which could take place inside the colon. Yiping W. Han identified the protein Fad2, exclusively encoded by *F. nucleatum* and *F. periodonticum*. Fad2, being not only adhesin but also an invasin, is essential for bacteria to first bind and then invade both host healthy and cancerous cells (Han, 2015). Other biomolecules presented on the surface of *F. nucleatum*, crucial in promoting development of CRC, are lipopolysaccharides (LPS), adhesin A (FadA) and fusobacterium autotransporter protein 2 (Fap2) (Coppenhagen-Glazer et al., 2015; Han, 2015). Thanks to modern molecular methods of detection such as qPCR, fluorescence quantitative (FQ)-PCR, droplet digital PCR (ddPCR) and fluorescence *in situ* hybridization (FISH), it was possible to establish that *F. nucleatum* was present in CRC neoplastic tissues (Marano et al., 2019). According to the studies conducted by Kensuke Yamamura, *F. nucleatum* occurred in 20% (4/20), 10% (2/20), and 45% (9/20) of esophageal, gastric, and CRC tissues, respectively (Yamamura et al., 2017). Different theories argue that *F. nucleatum* can be transmitted *via* hematogenous transmission, from its typical habitat in oral mouth to any part of human body, even that would be suitable for these bacteria to colonize, i.e., colon or even the pregnant uterus (Han, 2011). Another potentially cancerogenic oral pathogen is *P. gingivalis*, implicated in the pathogenesis of periodontitis, an inflammatory disease-causing atrophy of alveolar bone and gingiva inflammation. These hazardous bacteria can be responsible for promoting growth of other species of bacteria in its surrounding *via* various virulence factors, causing expansion of microbiome and leading to dysbiosis in that area (Xu et al., 2020). Furthermore, one of the most troublesome virulence factors is lipopolysaccharide (LPS), which is an endotoxin present as component of bacteria cell wall. It seems that LPS could be responsible for end-organ damage as well as sepsis, both representing outcomes of systemic inflammation caused by increased release of cytokines and shoutable factors, released as a hosts’ immunity response for the presence of LPS (Lien et al., 2000). Another important arguing issue is that *P. gingivalis* has two different types of fimbriae, minor fimbriae and major fimbriae, which allow bacteria to bond with hosts’ cells and invade them causing inflammatory reaction (Enersen et al., 2013). *P. gingivalis* is also able to produce the gingipains, a family of cysteine proteinase which cleave polypeptides at the C-terminal after lysine residue and hydrolyze peptide bonds. Gingipains may be able to disintegrate different extracellular matrix components, such as complement factors, immunoglobulins, cytokines as well as collagens, and thus enable *P. gingivalis* to avoid immunological reactions and clearance by the host, leading to the expansion of pathogenic microbiome and the induction to expansion of pathogenic microbiome and induction of its cancerogenic input (Curtis et al., 2001). Gingipains (and also others virulence factors related to *P. gingivalis*) affect the host immune system and consequently cause both local and systemic disorders (Bregaint et al., 2022). Recent study revealed that gingipains are essential virulence factors for the stimulation of the MAPK/ERK signaling pathway and thus encouraging the CRC cells proliferation (Mu et al., 2020). In another study it was also shown that gingipains induce COX-2 expression and production of PGE_2_ through their influence of human monocytes (i.e. activation of both MEK/ERK/AP-1 as well as IκB kinase/NF-κB p65 cascades) (Nakayama et al., 2022). It was revealed in many studies that *P. gingivalis* presence is enriched in colorectal mucosa as well as in the stool of CRC cancer patients (Koliarakis et al., 2019; Mu et al., 2020). Moreover, some studies proved that is a direct link between the abundance of bacteria in the oral cavity and in the gastrointestinal tumors microenvironment (Sobocki et al., 2021; Sobocki et al., 2022). It was also shown that *P. gingivalis in vitro* can adhere to CRC cells and then invade them only a few hours after administration. Therefore, *P.gingivalis* might promote CRC cells proliferation (Mu et al., 2020). Although the experimental data are still limited, there are indications that the role of *P.gingivalis* in CRC cancer prognosis is essential. In addition, oral hygiene and oral bacteria composition seem to influence the gut bacteria composition, cause dysbiosis, and in the end cancer promotion.

**Figure 1:**
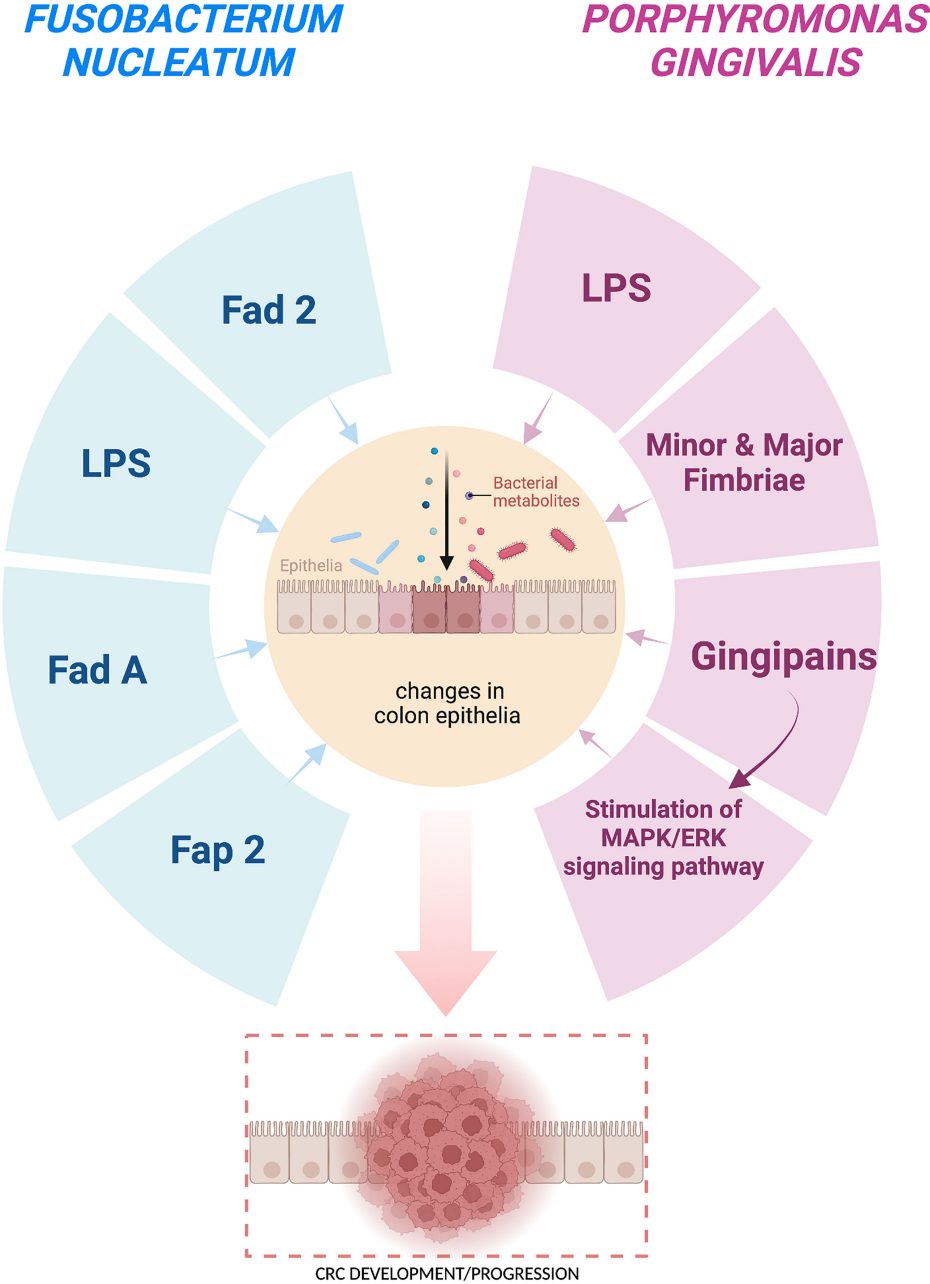
Carcinogenic effect of the virulence factors of *Fusobacterium nucleatum* and *Porphyromonas gingivalis* on the colon cells leading to CRC development/progression. Fad 2 – adhesin and invasion, LPS – lipopolysaccharides, Fad A – adhesin, Fap 2 – fusobacterium autotransporter protein 2, Gingipains – cysteine proteinases. This figure was created using *Biorender.com*.

The summary of virulence factors of *F. nucleatum* and *P. gingivalis* is presented in **Figure 1**.

## 5 Oral microbiome as a biomarker for CRC

Oral microbiome may be used as a biomarker for the detection of oral cancer. The results of Hayes et al. study revealed the link between oral microbiome and head and neck squamous cell cancer (HNSCC) (Hayes et al., 2018). It was observed that abundance of oral *Corynebacterium* and *Kingella* was related to the decrease of the risk of HNSCC. However, oral microbiome may be established as biomarker not only for local oral cancer, but also CRC. Promising insights into finding a non-invasive oral microbial biomarker for early detection of colon cancer detection have recently emerged.

In a Zhang et al. study, oral microbiome (analyzed from oral swab) was analyzed using 16S rRNA sequencing (Zhang et al., 2020). Patients with CRC (n=161), colorectal adenoma (n=34), and healthy volunteers (n=58) were enrolled. Interestingly, the composition of oral microbiome and its diversity were significantly different between the three groups of participants. The highest diversity was observed in patients with colorectal adenoma (Zhang et al., 2020). Furthermore, Flemer et al., analyzing microbiota profiling from samples of oral swaps as well as from stool and colonic mucosae from patients suffering from CRC (n=99), colorectal polyps (n=32), and healthy individuals (n=103) highlighted that CRC heterogeneity may be linked to microbiota types that either predispose to or provide resistance to the disease (Flemer et al., 2018). Therefore, profiling the oral microbiome could provide a non-invasive biomarker for CRC. For instance, the results showed that *Prevotella* spp. and *Streptococcus* spp. may differ in abundance depending on occurrence of CRC (Flemer et al., 2018).

Another promising research, conducted by Yang et al., showed that oral pathogens, especially *Prevotella intermedia* and *Treponema denticola*, were positively associated to increased risk of developing colorectal cancer (Yang et al., 2019). From the examination of mouth rinse samples, which were sequenced using the 16S rRNA gene, a correlation was found between abundance of specific taxa of oral microbiota and possibility of CRC occurrence. Wang et al. investigated whether *P. gingivalis*, an oral bacterium belonging to red complex bacteria, is capable to promote the development of colorectal carcinoma. Overall, 77 fecal samples were collected (22 without colorectal diseases, 32 with colorectal adenoma, 23 with colorectal cancer), and *P. gingivalis* had greater abundance between patients affected by CRC rather than adenoma as well as healthy people.

In conclusion, these findings indicate that high number of these pathogenic bacteria may not only be associated with higher risk of having CRC locally advanced but may also be a crucial biomarker that helps increasing the probability of early detection of this disease (Wang X et al., 2021).

## 6 Oral microbiota and treatment efficiency

Although the treatment of CRC is fairly well established, and the surgical resection remains the cornerstone of curative intent approach (which is sometimes combined with downstaging preoperative radiotherapy and systemic therapy) interest in tailored medicine has grown in recent years (Dekker et al., 2019). New therapeutic non-invasive methods, focusing on patient-specific and disease-specific predictive biomarkers, could support the preparation of patients for the surgical treatment, reduce the side effects of anti-cancer therapy and inhibit the progression of disease.

Currently, a considerable amount of literature on the influence of gut microbiota on different treatment modalities in CRC has been published. However, although the oral microbiota appears to play a similar role, the number of evidence and studies showing this is limited. A study from Dong et al. gave an insight into the mechanism of action (Dong et al., 2021). Studying mouse models of CRC, they revealed that buccal *F. nucleatum* may migrate to CRC locus impairing the efficiency of radiotherapy. The administration of metronidazole reduced this effect (Dong et al., 2021). In addition, it seems that oral microbiota may influence the composition of gut microbiota, affecting specifically the tumor microbiota, but not the microbes in adjacent tumor tissues. With this mechanism oral microbiota may play a role in radiation-induced intestinal injury (Dong et al., 2021). Interestingly, Gao et al. study showed that *F. nucleatum* may stimulate response to tumor and the efficiency of PD-L1 blockade in mice. This bacterium induced PD-L1 expression *via* activation of STING signaling, increasing of interferon gamma and the level of CD8+ tumor infiltrating lymphocytes (Gao et al., 2021). These results may indicate the need to adjust the applied treatment to oral and gut microbiota composition.

Another study conducted by Yoshihara et al. confirmed that the management of periodontal disease might cause the decrease of *F. nucleatum* levels in stool of patients who underwent successful treatment. On the contrary, this was not observed in the group of patients with the treatment failure (Yoshihara et al., 2021). This therapeutic strategy seems to be promising and its impact on clinical outcomes in CRC patients should be further investigated.

Definitive randomized control trials should deeply characterize the impact of oral microbiota on different treatment modalities efficiency in CRC, taking into consideration the important conclusions drawn by the studies above mentioned. The missing links (i.e., secreted metabolites) between oral microbiota and therapy efficiency should be revealed in order to discover potential targets for treatment.

## 7 Conclusions

Upon presented information, we established that human oral microbiome is, on one hand, vastly important to keep balance between host and coexisting bacteria, on the other hand, it may cause serious damage to general health even beyond area of oral cavity. The structure of oral microbiome may be influenced by various factors, such as age, sex, oral hygiene, and general state of health. Oral mouth is heavily occupied by many different bacterial species that inhabit it. Notably, some of those microbes are extremely pathogenic and because of their numerous virulence factors they may turn out to be cancerogenic.

The metabolites produced by pathogenic bacteria, i.e. *P. gingivalis* and *F. nucleatum* increase the likelihood of CRC development and may even stimulate its growth. Some data suggest that these bacteria may be used, in the future, as non-invasive predictive biomarkers, to detect cancer at different stages (putting the emphasis on earl stage), predict its development and ultimately take appropriate preventive measures before CRC occur. However, this method is strongly limited, among others, due to the fact that periodontal diseases (caused by these bacteria) are more common than CRC. Additionally, the establishment of microbial biomarkers is complicated process, which should regard multiple factors, such as type of taken material (for instance dental plaque, unstimulated saliva), methods of analysis, stage of CRC, age, condition of dentition, the level of oral hygiene, etc. The analysis of oral microbiome and metabolites produced by oral microorganisms in the context of CRC may be a future perspective for oncology. Therefore, it is recommended to introduce more effective cooperation between dentists, oncologists, and oncological surgeons. This interdisciplinary cooperation may open/strength searching for new options for CRC patients.

## Author contributions

All authors listed have made a substantial, direct, and intellectual contribution to the work and approved it for publication.
